# Key tips to providing a psychologically safe learning environment in the clinical setting

**DOI:** 10.1186/s12909-022-03892-9

**Published:** 2022-11-28

**Authors:** Philip Hardie, Roisin O’Donovan, Suzi Jarvis, Catherine Redmond

**Affiliations:** 1 School of Nursing, Midwifery and Health Systems, University College Dublin, Dublin, Ireland; 2 Centre for Positive Psychology and Health, RCSI, Dublin, Ireland; 3 Innovation Academy, University College Dublin, Dublin, Ireland

**Keywords:** Psychological Safety, Clinical Learning Environment, Nursing Education, Nursing Preceptorship

## Abstract

Having psychological safety embedded in preceptorship relationships facilitates positive interpersonal and educational experiences for students. Psychological safety refers to a student’s belief as to whether or not it is safe for them to take interpersonal risks, such as asking questions, sharing an idea for improvement or speaking up to maintain patient safety. Having psychological safety leads to collaboration, positive student learning experiences and effective patient care. This article presents key guidelines for preceptors to provide a psychologically safe learning environment for their students. Guidelines fall under four categories 1) before meeting students, 2) first meeting students, 3) continued relationship with students, and 4) general rules. These guidelines are informed by current literature on psychological safety and preceptorship and the author's clinical expertise in nursing preceptorship. We conceptualise psychological safety in a nursing preceptorship for preceptors to denote the experience of inclusivity, empowerment, and well-being of students within the social, cultural and physical clinical learning environment. A crucial attribute to cultivating a psychologically safe environment involves being an accessible and approachable preceptor.

## Background

Learning in the clinical environment is a cornerstone of healthcare education [1]. An effective and supportive clinical learning environment (CLE) provides trainee healthcare professionals with an opportunity to develop professional behaviours, link the theoretical aspect of their studies with clinical practice and strongly influence students' achievement of their programme learning outcomes [2, 3].

Central to the learning in the CLE in nursing education is preceptorship. Preceptorship has been defined as: *'an approach to the teaching and learning process within the context of the practice setting which allows students to develop self-confidence while increasing their competence as they become socialised into the profession of nursing'* [4, pg.259]. A fundamental component of preceptorship is the 'preceptorship relationship', a triadic professional relationship between the preceptor (experienced staff nurse) and the preceptee (nursing student), centred on the delivery of patient care within a clinical environment [5]. With 50% of the undergraduate nursing curriculum in a higher education institution and the other 50% in the clinical environment, preceptors play a pivotal role in educating student nurses in the clinical environment. It is widely acknowledged that the quality of the preceptorship relationship can significantly influence the student's integration into the nursing profession and the clinical environment and can affect the student's professional development and delivery of patient care [6].

A productive clinical learning environment for students depends on positive interpersonal relations. When preceptors take an interest in the supervision of students, are easy to approach, and engage and collaborate with students and patients, it positively influences the students learning experience [7]. Nursing students have also reported that when preceptors create an atmosphere of respect and recognition of the students' work, it positively impacts their learning, creating an inclusive and psychologically safe learning experience [1].

### What is psychological safety?

*Psychological safety* was initially defined as a "*shared belief that the team is safe for interpersonal risk-taking*" [8]. These interpersonal risks include open communication, voicing concerns, asking questions and seeking feedback without fear of judgment [9]. In other words, psychologically safe environments allow individuals to be their authentic selves, improve individuals’ well-being, and reduce work-related stress, leading to increased engagement and learning. Within hierarchical healthcare teams, individuals in more junior positions often find it difficult to speak up against the status quo and this reduces opportunities for collaborative learning [10]. Therefore, psychological safety required that deliberate efforts are made to reduce the professional hierarchy often observed in healthcare teams and clinical education [11].

Seminal work by behavioural psychologist Amy Edmondson [8, 10] established that when leadership and interpersonal climate support candour, humility, learning from error, and an appreciation of input from everyone on the team, psychological safety can occur. Moreover, essential work carried out by Clark [12] in the field of psychological safety illustrates four stages of psychological safety; 1) feel included; inclusion safety, (2) feel safe to learn; learner safety, (3) feel safe to contribute; contributor safety and (4) feel safe to challenge the status quo; challenger safety. Clark [12] argues the four stages of psychological safety are a universal pattern that reflects the natural progression of human needs in social settings. In the context of learning, Clark [12] highlights the importance of learner safety in asking questions, giving, and receiving feedback, experimenting, and making mistakes when learning. The innovative and seminal work of Edmondson [8, 10] & Clark [12] has pioneered effective practices to creating psychological safety.

## Methods

We provide an argument that a preceptor's ability to provide a psychologically safe learning environment for their students can significantly impact the students learning experience. Therefore, the authors set out to examine the available literature on psychological safety in a clinical learning environment, particularly in the context of a nursing preceptorship. This review aimed to establish what is known about psychological safety in a clinical learning environment. From this evidence base the authors set out to establish key tips for preceptors to create a psychologically safe learning environment for students in a nursing preceptorship review sought to address the following research question:

- What is known about psychological safety in a clinical learning environment?

A comprehensive literature search was employed to inform the development of the innovative tips for providing a psychologically safe learning environment in the clinical setting.

The search was conducted within the following databases:

- CINAHL, PsycINFO and PubMed.

The search strategy was deployed in Jun 2022 to identify the most up-to-date literature. See Table 1:

**Table 1:** Key search terms for literature review

Preceptor (or) Mentor (or) Supervisor (or) Clinical learning environment (or)Practice placements (or) clinical education (or) nursing education (or) medicine education
AND Psychological* safety (or) safe learning environment

Studies eligible for inclusion were peer-reviewed or opinion pieces from any country, published between 2000 and 2022 and explored the development, implementation and/or evaluation of interventions relevant to psychological safety in a clinical learning environment. Given the limited number of interventions targeting psychological safety in nursing education, the search was expanded to include medical education. The grey literature was examined to identify professional guidance on learning in the clinical environment.

Studies were excluded if they were not available in English or if they reported on interventions conducted outside healthcare settings.

The initial electronic database searches retrieved 1127 references. Search results were uploaded to Mendeley reference management system (https://www.mendeley.com) to aid screening. Duplicates were removed (*n* = 371). The lead author carried out title and abstract screening. Common reason for exclusion (*n* = 700) was the focus was not on psychological safety in a clinical education setting. 56 full screen papers resulted in inclusion of two review papers, two case studies, five empirical papers, three commentary papers and three professional guidance documents (*n* = 13).

This review used a deductive thematic synthesis (Braun & Clarke, 2022) to combine the studies examining psychological safety in a clinical learning environment to a) examine psychological safety in clinical education b) examine psychological safety in the context of a nursing preceptorship. Furthermore, a content analysis was carried out on empirical research and professional guidance documents to identify universal themes for creating psychological safety so that they could then be applied to a nursing preceptorship scenario based on the authors clinical expertise in nursing preceptorship.

### Psychological safety in clinical education

There is growing body of conceptual and empirical work focusing on understanding the nature of psychological safety, identifying factors that contribute to it, and examining its implications for individuals, teams, and organizations within healthcare clinical education. According to Edmondson et al. [10], psychological safety is critical in enabling individuals to overcome barriers to learning in interpersonally challenging environments. In a psychologically safe clinical learning environment, students feel safe to take risks, make mistakes, and ask for help or support when needed. Furthermore, this creates a safe environment where students can identify what skills they lack and wish to improve without judgment. The feeling of safety is an essential element in learning environments, where students are expected to take risks [13].

Psychological safety has been seen as an important element underpinning learning behaviours. Current literature reports that when students feel psychologically unsafe, they tend to refrain from doing or saying something that might suggest incompetence [14]. An unsafe psychological environment can also raise anxiety and lead to avoidance and disengagement of students' clinical education and patient care [15, 16]. Students can find it challenging to think clearly, retain information or perform clinical skills they previously felt competent in [14]. Psychological safety also plays a vital role in students speaking up for patient safety or questioning nurses when they observe inconsistencies between what is taught at university and what is performed in practice [17].

Psychologically safe learning environments permit students to seek clarification or help to answer questions or demonstrate skills workout fear of judgement or humiliation [18]. It is important to highlight that while preceptors should create an environment where students can make mistakes without being judged or punished, they must ensure patient safety throughout their practice placement [19]. Having a psychologically safe environment does not mean that there is a lack of accountability [10]. In fact, accountability and psychological safety are both required in order to students to operate within the “learning zone” [10]. Furthermore, students are expected to engage at a supernumerary level, adhering to established professional guidelines and code of conduct and only performing tasks they have learned and are competent to perform [20].

To contextualise the principles of psychological safety and learning in the clinical environment, it is important to consider Kolb's experiential learning theory. Due to its interpersonal and practical nature, experiential learning is critical for nursing skill development [21]. According to Kolb's experiential learning theory, learning is a process through which experience is transformed into new knowledge [22]. Kolb's learning cycle involves four stages: concrete learning, reflective observation, abstract conceptualisation and active experimentation [23]. More recently, Kolb and Kolb [24] acknowledge the importance of psychological safety in creating an effective learning space. Under this concept, the individuality of learners is faced and embraced by encouraging differences of expression. The learner and instructor can engage in fearless communication, i.e., express how they feel or their level of knowledge without fear of being reprimanded. In the absence of psychological safety, people do not share their unique knowledge, thus posing a threat to learning [8]. Furthermore, speaking to Kolb's experiential learning cycle principles, a factor in creating psychological safety in the clinical learning environment is identifying the learners' preferred learning style. Kolb postulated that a learning experience is maximised when the learner and facilitator consciously try to engage with the learner's learning style [25].

Psychological safety principles are also related to Maslow's (1943) hierarchy of needs. Maslow's (1943) hierarchy of needs [26]. Maslow highlights that our actions are motivated by particular psychological needs; 1) psychological needs, 2) safety needs, 3) love and belonging needs, 4) esteem needs, and 5) self-actualisation needs. A student can only move to address the higher-level needs when their basic needs are fulfilled. For example, once students feel safe, they seek social interaction and feel a sense of belonging followed by respect and achievement. Therefore, preceptors must allocate an appropriate level of time to establish psychological safety and maintain positive interpersonal relations within the preceptorship relationship to create a safe and inclusive learning environment for students from the outset of the professional relationship and throughout.

The principles of psychological safety also speak to Mezirow’s theory of transformative learning [27]. Mezirow & Cranton [28,pg74] defined transformative learning as “*the process of using prior interpretation to construe a new or revised interpretation of the meaning of one’s experience as a guide to future action*”. Constructs of critical reflection, dialogue, and experience are considered central to transformative learning theory [25]. Psychological safety creates an environment where the learner is comfortable embracing feelings of uncertainty associated with learning new skills in the clinical environment. Psychological safety permits the learner to feel safe to openly admit if they do not know something or are unsure of how to complete a particular skill, thus facilitating a positive learning experience and, most likely, a productive learning experience for the learner. Like Kolb’s experiential learning theory, critical reflection also plays a central role in Mezirow’s transformative learning theory. Psychological safety creates a safe environment for learners to critically reflect and discuss experiences within the clinical learning environment to generate a new understanding of the experience.

### Psychological safety in nursing preceptorship

As discussed in the previous section psychological safety plays a central role in education in the clinical setting. The authors argue that psychological safety also plays a crucial role in a nursing preceptorship, where students' clinical placement outcomes rely heavily on the interpersonal dynamic and assessment associated with the preceptorship relationship. Preceptors are responsible for teaching and assessing students using a competency assessment criteria [29, 30]. Preceptors are also responsible for ensuring they provide a supportive culture for students [29]. Students’ perception of psychological safety is likely to mediate the relationship between preceptor-to-student interactions and students’ perceived quality of their learning experience [18]. The current literature suggests a lack of guidelines for preceptors to address the psychological safety needs of their students. Current guidelines for psychological safety are typically focused at an organisational and team level [31]. The guidelines presented in the next section are targeted at individual preceptors who aim to provide a psychologically safe learning environment for their students.

### Guidelines to providing psychologically safe learning environments for students

In the following section, the authors will describe key recommendations to fostering psychological safety for students in clinical learning environments based on Edmondson's [8, 10] and Clarke's [12] seminal work in psychological safety, the available literature on psychological safety in clinical education, international professional guidance documents [29, 32, 33] and the author's expertise in the field of preceptorship. Table 2 below presents guidelines for creating a psychologically safe learning environment in the context of a nursing preceptorship under four categories 1) before meeting students, 2)first meeting students, 3) continued relationship with students and 4) general rules. It describes each of the individual steps, providing a rationale and example for each.

**Table 2:** Guidelines to providing a psychologically safe learning environment for students

These guidelines are designed to help you as a preceptor apply a standardised approach to providing a psychologically safe learning environment for your students.		
Prior to Meeting Student		
Steps	Rationale	Examples
Formulate a set of questions specific to your area of nursing	This will help activate the learning process for students	Choose questions suitable to your clinical area.
Identify and reflect on any unconscious negative bias (towards students), so you can act to remove them.	Unconscious bias happens when we allow our attitudes, feelings, stereotypes, or beliefs to impact our judgement or understanding of students. Unconscious bias also affects how you communicate. It can affect the morale and the overall experience of the students. They may feel alienated and be less likely to make their ideas heard. Your nonverbal communication can give away your underlying bias, resulting in students feeling unwanted and a loss of confidence.	Stereotyping students, e.g., all students are not interested in learning, ageism, gender bias, bias relating to students’ stage of their programme (1st years have little knowledge or skills, fourth-years should be able to do everything).
**Initial Meeting Student**		
Steps	Rationale	Examples
Introduce yourself at the first opportunity	Be proactive to introduce yourself. Establishing *yourself* as an open, friendly, and professional individual will “break the ice” and display warmth, acceptance, and a sense of inclusion, encouraging the student to engage with you.	“Hello, my name is ....... I will be your preceptor. You are very welcome”.
Identify how the student likes to be addressed and how to pronounce their name	Calling a person by their preferred name shows respect. It helps provide an empowering, safe, and non-discriminatory educational and work environment. A name is an extremely important part of a person’s identity; mispronunciation of their name can cause insult, signalling to the student that they are less important and less valued.	“It is so lovely to talk with you, (insert name)- Do you prefer (insert name) or do you go by another name? “What do you like to be called"? “I want to make sure that I say your name correctly. Could you pronounce your name for me, please”?
Define and communicate the purpose of the professional relationship and your role as their preceptor	To feel a part of the preceptorship relationship, the student must understand the purpose of the relationship, your role as their preceptor, and how it will work. It creates an open and transparent relationship with a clear understanding of the purpose and role in the relationship.	“I will be working with you over the next few weeks; I am here to work alongside you in providing patient care, teach you, provide you feedback and assess your level of competency throughout your placement. I am here to answer any questions you may have and help you achieve your learning goals for the placement ”.
Build a rapport by sharing appropriate background and experiences about yourself; Ask students questions to discover their interests.	Providing students with a summary of your experience to date and appropriate personal information helps build a friendly and inviting rapport. Asking students about their experiences and personal interests helps create a common ground.	“I have worked on a surgical ward for 10 yrs. Prior to working here.....* I was drawn to nursing because ........etc* Tell me a little about yourself? What experience have you had to date? Do you have any hobbies”
Collaborate with the student to set concrete and time-bound learning goals	Students do better when they feel in control of their learning. Working with your students to set reasonable but achievable goals in the placement timeframe drives their learning and makes them feel they are leading their learning. Reassuring students that learning takes place over a period of time aids their ability in setting realistic expectations.	“What would you like to learn on this placement?” “I would like you to achieve the following......how does that sound to you?” “I feel you can achieve the following learning objectives during your placement ...... how does that sound to you? Is there anything else you would like to learn? “
Communicate a vision of the student's potential	Helping the student see what they can do can stimulate the student’s motivation to learn.	“I think that you will get on great during your placement, you have a great knowledge base starting, and you are extremely capable of passing this placement.” Alternatively, more specific, “I think you will be able to complete a full pre-op assessment by the end of the week. You are already doing most of it on your own. Keep up the good work!”.
Identify with your students each other’s strengths and weaknesses in areas of nursing	Identifying and utilising your strengths has been linked to an elevated sense of vitality and motivation, increased probability of achieving goals, and a stronger sense of direction. It is also linked to higher self-confidence, engagement, and productivity. Actively and creatively reflecting and problem-solving around your strengths and weaknesses can motivate you to improve. It also builds team cohesion and builds trust as you help each other improve areas of weakness.	Identifying each other’s strengths and weaknesses; for example, being good at medications and communicating with patients, requires improvement in managing patient workload and dressings. “ What do you feel are your strongest and weakest areas of clinical practice?” “What aspects of nursing do you feel you are good at?” “What areas do you feel you could improve on?”
Identity students’ triggers for stress	It is crucial to identify the triggers that might affect a student’s academic, social, emotional, and behavioural status during their practice placements. Academic stressors, interpersonal stressors, and environmental stressors influence students’ health and feeling of psychological safety during their practice placements.	For example, a student could state, if I do not hand over completely before we finish, I lay in bed at night wondering did I do this, did I sign that form etc. To avoid this, ensure to include students in the handover process, thank them for their days' work, and enjoy their evening. Alternatively, another student may find speaking to a packed room in handover extremely stressful. Work with the student to overcome this, maybe start with one patient and then build it up to complete handover.
**Continued Student Relationship**		
**Steps**	**Rationale**	**Examples**
Conduct frequent touchpoints	Frequent interactions are more effective than long, infrequent ones. Frequency builds and strengthens connections.	Have short daily one to one meetings to provide oral feedback to students instead of 2-to 3 long formal NCAD meetings. Touch base with your student throughout the day, agree on 3-4 check-ins per shift, keep communication open and create a safe environment to ask questions.
Model a positive mindset to learning. Get to know their preferred learning style.	This makes learning a collaboration (you will learn from the student also).	Share something new that you learned to demonstrate that learning is ongoing and that you do not know everything. Establishing their learning style will also put the student at ease, creating a psychologically safe environment.
Get to know their preferred working style.	Encourages effective teamwork and reduces the risk of conflict.	What way shall we plan our day? Do you prefer to take four patients each, or shall we team nurse and break up the workload for all eight patients?
Share past mistakes.	Creates a culture to discuss mistakes without blame or repercussions. Reduces students' stress if an error does occur, thus improving their learning experience. Sharing mistakes also creates vulnerability, thus further connecting student and nurse relationships.	Stating that every nurse is likely to make a mistake at some point in their career, providing an example.
If a student makes a mistake, do not judge or reprimand them. Reinforce their effort into achieving the skill, highlight how it can be improved, and ask how you can help them achieve this.	Making mistakes during placements should be a learning opportunity to improve your students' practice. Reinforcing their effort and highlighting areas for improvement will help create a non-judgemental, fear of failure teaching environment for your students.	“Well done on administering your first sub-cut injection. You did a great job following the ten rights and putting the patient at ease; make sure next time you bring the sharps bin right over to the patient; you increase the risk of a needle stick injury if you leave it on the bedside table. Overall, well done. You did a great job”.
Express gratitude and appreciation towards students for their input to the team	This further helps establish an inclusive and appreciative environment where the student feels valued and part of the team, resulting in a positive clinical learning environment.	Sometimes, something as simple as a 'thank you goes a long way! “Thank you for all your work today. I would not have managed without you. We work well together as a team. I appreciated all the work you did”. “Your compassion, optimism and kindness to your patients do not go unnoticed....”
Demonstrate learning empathy	Understanding and stating that you know what one student might find easy another may struggle with. You also appreciate that students have a lot to remember and that you remember being a student and being worried and stressed will help create a psychologically safe learning environment for the student as your level of understanding and empathy will put the student at ease and build their trust in the relationship.	“I want you to know that I remember what it is like to be a student, so please do not be afraid to ask me questions or show you something again if you do not get it the first time. I know how students feel overwhelmed and stressed at times. Let me know if you feel like that in any way, and we will work together to sort it out”.
Admit when you do not know something	Being open and admitting you do not know something creates trust between you and your student, creating a stronger open and transparent relationship and, therefore, a positive learning environment.	“I should know what this medication is for, but to be honest, I cannot remember. Let us look it up together so we can learn together”.
Ask the student for feedback and embrace it	Asking students for feedback creates a bidirectional feedback opportunity that helps reduce hierarchical relationships creating an equal relationship where both parties’ views are appreciated and an opportunity to reflect and improve for future practice.	“Did you learn everything you feel you set out to learn on this placement? Is there anything I could have done differently as your preceptor to better your clinical placement? Did you feel the frequency and level of feedback you received were suitable to help you achieve your learning goals? “
Offer support to a student you can see is struggling	Offering support creates psychological safety as it shows the student that you care about the student and their education.	Talk to your students. What are they struggling with outside of the classroom? Are they overwhelmed by work or family commitments? Perhaps they could use some coaching around time management. Or a more difficult question involves asking for an honest answer about how much effort they are putting into studying
**General Rules**		
**Steps**	**Rationale**	**Examples**
Physically face students when talking to them, make eye contact	Making eye contact helps both preceptor and student or patient focus on the conversation, and reading facial expressions can improve understanding, thus significantly improving communication and personal connections.	Please do not write your notes as you speak to your student. Please stop what you are doing, face them and make eye contact.
Use appropriate humour	Humour is a subjective emotional response, which has multi-dimensional value in preceptorship relationships, including bonding on a personal level, developing team cohesion, the release of dopamine that can promote positive thinking, creativity and reduce stress hormones increasing a person's ability to cope, enhance memory, increase self-esteem, optimism, and vigour. Humour can be a helpful coping strategy and help reframe and defuse a problem by putting it into perspective. However, humour is inappropriate in psychological crises and emergencies.	Appropriate humour can be a positive distraction, for example, when undertaking a clinical procedure such as a wound dressing or personal care, putting the student and the patient at ease.
Be aware of your nonverbal cues	Be careful that you do not send nonverbal cues that communicate exclusion.	Eye rolling, fidgeting, drumming your fingers, looking bored or away or allowing yourself to be distracted in the middle of a conversation.
Never compare your student to another student	When preceptors compare one student to other students, they typically compare what they lack to another student. This is not productive as it can be a biased comparison and will not help the current student achieve their goals.	“My last 4th-year student had no problem inserting an NG tube. Why is it that you cannot? I had expected you could complete this simple skill”.
Avoid exclusive patterns of social interaction (i.e., just with qualified nurses) as this will exclude students.	This creates invisible sociable barriers and a sense of exclusion. Be inclusive of the entire team.	Do you tend to interact with the same people outside what is required of your role? For example, do you always go to breakfast with the same nurses? Would you invite your student on the break with you? Avoiding this pattern of social exclusion leaves the invite open to all.

Lyman and colleagues’ recent study [18] examining new graduate nurses' experiences of psychological safety found four main themes emerged, including: building credibility (building the trust of others in their capabilities), making personal connections, feeling supported and seeking safety. Similarly, a more recent concept analysis of the application of psychological safety to health care found five attributes: perceptions of the consequences of taking interpersonal risks, strong interpersonal relationships, group-level phenomenon, safe work environment for taking interpersonal risks and non-punitive culture to influence psychological safety [34]. In addition, a systematic review of factors that enable psychological safety in healthcare teams found five broader themes: priority for patient safety, improvement of learning orientation, support, familiarity with colleagues, status, hierarchy and inclusiveness and individual differences impacted psychological safety [11].

Collectively the evidence above suggests the critical role making strong interpersonal connections plays in initiating a psychologically safe environment for students. It lets preceptors connect with their students beyond surface-level greetings or small talk. Identifying students preferred names creates an atmosphere of respect [35]. On the other hand, a lack of openness or personal engagement from preceptors has resulted in negative learning experiences for students [36]. Therefore, the authors recommend to *“Identify how the student likes to be addressed and how to pronounce their name”* and *“Build a rapport by sharing appropriate background and experiences about yourself; Ask students questions to discover their interests”* (Table 2)*.*

Moreover, international guidance documents for nurses on learning in the clinical environment [29, 30, 37] recommend that all students are made aware of the supports and opportunities available to them within the clinical environment. This includes students clearly understanding the purpose of the professional relationship preceptorship offers, your role as their preceptor, and how it will work. *Therefore, the authors recommend “Define and communicate the purpose of the professional relationship and your role as their preceptor”*(Table 2).

Inclusivity is another fundamental component of creating a psychologically safe learning environment [10, 20]. Without addressing social and cultural aspects of the clinical learning environment, there is a risk that students may feel excluded from the ward resulting in students feeling a lack of belonging or value to the team [20]. Social acceptance and feeling part of the team have led to motivation to provide care and clinical learning amongst students [38]. Therefore, the authors recommend to “*Express gratitude and appreciation towards students for their input to the team*” and “*Avoid exclusive patterns of social interaction*” (Table 2).

Trust is a crucial aspect of psychological safety. Trust forms the foundation of psychological safety, acknowledging vulnerabilities and allowing reciprocal expectations and beliefs to rely on each other during the teaching and learning process [33]. Preceptors must create a trusting atmosphere for their students. Therefore, the authors recommend” Identity with your students each other’s strengths and weaknesses in areas of nursing” and “Demonstrate learning empathy”.

Unintentional bias is another factor that can result in a psychologically unsafe environment for students [39]. Preceptors must examine any bias or stereotypes they may have towards students to remove barriers to effectively communicating and engaging with students to promote belongingness in the preceptorship relationship. Therefore, the authors recommend to “*Identify and reflect on any unconscious negative bias (towards students), so you can act to remove them*” (Table 2).

Setting clear expectations and learning objectives is also a valued action to create a psychologically safe learning environment for students [14]. When clear learning objectives are set collaboratively, it is shown to positively influence the students learning experience [40]. Therefore, the authors recommend to “*Communicate a vision of the student's potential*” and “*Collaborate with their student to set concrete and time-bound learning goals*” (Table 2).

As previously outlined Edmondson [10] established creating an interpersonal climate that supports openness and honesty is central to psychological safety and learning. Therefore, in the context of providing a psychologically safe environments for students, the authors recommend preceptors take the following steps: “Identify with your students each other’s strengths and weaknesses in areas of nursing”, “Admit when you do not know something”, “Share past mistakes” (Table 2)*.*

Equally, Clarke [12] established the importance of learner safety and psychological safety in one’s ability to ask questions. This speaks both to the preceptor and student in a nursing preceptorship. Therefore, the authors recommend that preceptors ask structured questions to spark students learning; “*Formulate a set of questions specific to your area of nursing*”. The authors also recommend “*conducting frequent touchpoints*” to create an atmosphere where students feel safe to ask questions (Table 2).

Another critical factor in creating a psychically safe learning environment is engaging students in collaborative and bidirectional feedback (30; 31,15]. Bidirectional feedback creates a collaborative environment and demonstrates a mutual respect [41]. Psychological safety makes it more likely that students will engage in safe clinical practice and collaborative discussions with their preceptors, thus supporting learning and helping develop a sense of teamwork and belonging. Therefore, the authors recommend to “*ask the student for feedback and embrace it*”. Furthermore Clarke [12] established the importance of making mistakes when learning and the importance of creating an environment in which there is fear or reprimand. Therefore, the authors recommend to “If a student makes a mistake, do not judge or reprimand them. Reinforce their effort into achieving the skill, highlight how it can be improved, and ask how you can help them achieve this” (Table 2).

## Discussion

This paper outlines guidelines for preceptors to provide a psychologically safe learning environment for their students (Table 2). These guidelines were recently incorporated into an innovative preceptorship education programme accredited by the Nursing and Midwifery Board of Ireland (NMBI), focusing on developing preceptors' interpersonal and communication skills relevant to student teaching and learning [32]. Implementing these guidelines could help preceptors establish a standardised approach to providing a psychologically safe learning environment for their students. More research is required to determine the impact of implementing these guidelines from the perspective of preceptors and the perspective of students on creating a psychologically safe learning environment following completion of the new educational programme.

## Conclusion

Psychological safety is an essential component of an effective nursing preceptorship. It is evident from the available literature that psychological safety plays an essential role in ensuring a positive and productive learning experience for students in the clinical learning environment. It is vital that students feel safe to ask questions and verbalise areas of clinical practice that require improvement without fear of judgment or repercussions for their clinical assessment documents. Key attributes preceptors must display are openness, inclusivity, effective interpersonal and communication skills, and a collaborative approach to creating a psychologically safe learning environment for students. Learning in a clinical context is foundational in training all health professionals. While the focus of this paper was applied to creating a psychologically safe learning environment within a nursing preceptorship, the authors argue that these principles and guidelines could be adapted and applied to suit different health professional training programmes.

### Take-home message

- Psychologically safe learning environments are essential for optimal learning in a nursing preceptorship.
- Students' learning is enhanced when students perceive a psychologically safe learning environment in the clinical setting.
- Preceptors defining attributes to creating a psychologically safe learning environment for students include openness, inclusivity, effective interpersonal and communication skills and collaboration with nursing students
- Preceptors must allocate an appropriate amount of time to establish and maintain psychological safety within the preceptorship relationship.

## Data Availability

Not applicable. Ethics approval and consent to participate. Not applicable.
